# Long term effects of cocaine on the heart assessed by cardiovascular magnetic resonance at 3T

**DOI:** 10.1186/1532-429X-16-26

**Published:** 2014-04-23

**Authors:** Alicia M Maceira, Carmen Ripoll, Juan Cosin-Sales, Begoña Igual, Mirella Gavilan, Jose Salazar, Vicente Belloch, Dudley J Pennell

**Affiliations:** 1 Cardiac Imaging Unit, ERESA Medical Center, Valencia, RIC, Spain; 2 Addictions Treatment Unit of Campanar, La Fe Hospital, Valencia, Spain; 3 Department of Cardiology, Hospital Arnau de Vilanova, Valencia, Spain; 4 Department of Psychiatry, Consorcio Hospital General, Valencia, CIBERSAM, Spain; 5 NIHR Cardiovascular Biomedical Research Unit, Royal Brompton Hospital & Imperial College, London, UK

**Keywords:** Cocaine, Magnetic resonance, Heart failure

## Abstract

**Background:**

Cocaine is an addictive, sympathomimetic drug with potentially lethal effects. The prevalence and features of cocaine cardiotoxicity are not well known. We aimed to assess these effects using a comprehensive cardiovascular magnetic resonance (CMR) protocol in a large group of asymptomatic cocaine users.

**Methods:**

Consecutive (n = 94, 81 males, 36.6 ±7 years), non-selected, cocaine abusers were recruited and had a medical history, examination, ECG, blood test and CMR. The CMR study included measurement of left and right ventricular (LV, RV) dimensions and ejection fraction (EF), sequences for detection of myocardial oedema and late gadolinium enhancement (LGE). Images were compared to a cohort of healthy controls.

**Results:**

Years of regular cocaine use were 13.9 ± 9. When compared to the age-matched healthy cohort, the cocaine abusers had increased LV end-systolic volume, LV mass index and RV end-systolic volume, with decreased LVEF and RVEF. No subject had myocardial oedema, but 30% had myocardial LGE indicating myocardial damage.

**Conclusions:**

CMR detected cardiovascular disease in 71% of this cohort of consecutive asymptomatic cocaine abusers and mean duration of abuse was related to probability of LV systolic dysfunction.

## Background

Cocaine is a highly addictive sympathomimetic drug with potentially lethal effects [1,2]. The annual prevalence of cocaine use is 0.3% to 0.5% of the world population aged 15-64 [3], corresponding to roughly 18 million people. Standardised mortality ratios suggest that mortality is 4-8 times higher among cocaine users than age and gender peers in the general population [4].

Cocaine cardiotoxicity appears multifactorial through several mechanisms involving the development of ischemia and infarction [5], ventricular hypertrophy [6], systolic dysfunction [7], arrhythmias [8], endocarditis [9], and aortic pathology [10]. Cocaine is usually consumed along with other substances, mainly ethanol and tobacco, which can exacerbate the deleterious effects of cocaine [11].

The majority of studies that have assessed the prevalence and pattern of cardiovascular involvement due to cocaine abuse are autopsy series [12] or retrospective studies that focus on patients who presented with a particular disease or symptom in whom cocaine use was retrospectively evaluated. There are limited data on the effect of cocaine in asymptomatic users [13], and more evidence is needed. Moreover, the majority of the published studies have been done with echocardiography, include a modest sample size, and do not take into account pattern and time course of cocaine use and concomitant use of other substances.

Cardiovascular magnetic resonance (CMR) is the current gold standard technique for the assessment of ventricular dimensions and function, with high accuracy and reproducibility and reference values established for both the left [14] and right ventricles [15]. CMR can provide a comprehensive evaluation of the cardiovascular system and has a unique capability for tissue characterization, which has been applied to the assessment of several cardiomyopathies [16-18].

Therefore, we aimed to prospectively assess the presence and pattern of cardiovascular involvement due to cocaine abuse in consecutive, asymptomatic cocaine abusers (CA) with a comprehensive CMR protocol, adjusting for the effect of other concomitant variables such as tobacco or alcohol abuse.

## Methods

This was a prospective study for which we recruited consecutive asymptomatic CA (males and females, 18-60 years) who fulfilled the criteria for cocaine addiction or abuse [19], who were attending a rehabilitation clinic for the first time and who had no exclusion criteria (Table 1). The maximum time interval allowed between the last cocaine use and the CMR study was 3 months. All the eligible CA were informed about the study and invited to participate. The study was approved by the institutional ethics committee (IRB of Hospital Arnau de Vilanova, Valencia, Spain) and all patients gave their informed consent. As a reference for normality, we used data obtained in a group of gender and age matched healthy volunteers that have been previously published [14].

**Table 1:** Exclusion criteria

• Diabetes mellitus, defined as plasma glucose >126 mg/dL or use of antidiabetic drugs	
• Severe chronic renal insufficiency (GFR < 30 mL/min/1.73 m^2^ calculated with the MDRD equation)	
• AIDS diagnosis, conditions with short life-expectancy	
• Consumption of design drugs within the last 5 years*	
• Allergy to iodine or gadolinium based contrast agents	
• Claustrophobia	
• Metallic implants (eg pacemakers, defibrilators)	
• Acute psychotic attack	

*Design drugs investigated were METH (methamphetamine, also called speed, crystal meth), MDA (3,4- methylenedioxyamphetamine, also known as love drug), MDMA (3,4-methylenedioxy-N-methylamphetamine, also known as ecstasy) and MDEA / MDE (3,4- methylenedioxy –N-ethylamphetamine, also called Eve).

### Medical history

Data from the patients’ medical records were collected. Hypertension and dyslipidemia were defined according to the current guidelines [20,21]. Patients were classified as non-smoker, ex-smoker (1 month to three years after quitting smoking), and smoker. Overweight and obesity were defined as body mass index of 25-29.9 kg/m^2^ and ≥ 30 kg/m^2^, respectively. Diabetes mellitus was defined according to the ADA 2008 criteria [22]; from 2010 onwards HbA1C was also considered [23]. Family history of coronary heart disease and pharmacological treatment at the time of the study entry were investigated.

### Psychiatric evaluation

Cocaine abuse or dependence was ascertained using the Structured Clinical Interview for Diagnostic and Statistical Manual of Mental Disorders, Fourth Edition (DSM-IV) [24] and by documenting a cocaine-positive urine screening test at the time of enrolment. Lifetime use was assessed with the Life Severity Index for cocaine and we recorded the route of administration (nasal inhalation, smoked, intravenous), frequency of use during the three months prior to CMR, date of last cocaine use and years of regular cocaine use [25,26]. Finally, concomitant use of alcohol and tobacco were investigated with validated standardized scales [24,27]. Years of alcohol use, current number of units of standard drinks, smoking history as well as number of cigarettes were also recorded.

### Physical examination and laboratory tests

These included recording of weight, height, heart rate, blood pressure and a cardiovascular routine evaluation. Blood sampling was performed for blood count, usCRP, fasting glucose levels, lipid profile, GOT, GPT, GGT and fibrinogen. Urine cocaine screening tests for the cocaine metabolite benzoylecgonine (Multicassette Drug Test, SureScreen Diagnostics Ltd, United Kingdom) were done. A rest electrocardiogram was obtained prior to the CMR scan.

### CMR protocol

The study was done in a 3 T scanner (Achieva 3 T TX, Philips, The Netherlands). Images were obtained using a 32-channel surface array coil with multitransmit parallel radiofrequency transmission. Multislice dark blood and bright blood single shot sequences were acquired. High resolution balanced SSFP cine sequences with retrospective gating were obtained in the usual cardiac views as well as in an oblique sagittal plane of the aorta. Typical parameters were TR = 3.2 ms, TE = 1.49 ms, field of view = 360 mm × 290 mm, matrix size = 256 × 224 (in-plane resolution 1.4 mm × 1.3 mm), slice thickness 7 mm, slice gap 3 mm, flip angle 30°, temporal resolution 28 ms, 40 phases. STIR sequences in end-expiration were acquired in the same views (TR/TE/TI/ matrix/slice thickness = 1800 ms/75 ms/190 ms/ 220 × 180/7 mm). High resolution transverse multislice T2 FSE sequences of the thoracic aorta were obtained (TR/TE/matrix/slice thickness/gap = 2000 ms/85 ms/340 × 290/7 mm/3 mm).

In the first 48 subjects, dipyridamole (0.84 mg/Kg in 6 minutes) stress myocardial perfusion images were acquired by using a saturation prepared gradient-echo sequence (TR/TE/flip angle/turbo factor/matrix/spatial resolution of 2.8/0.9/18°/59/128 × 128/2.5 × 2.5 × 8 mm), in three ventricular short-axis sections during gadolinium bolus administration (0.1 mmol/Kg). Images were also acquired at rest at the end of the protocol. In the subsequent 46 subjects the same myocardial perfusion sequence was acquired at rest.

Finally, segmented inversion-recovery sequences (IR-TFE) were acquired for detection of late gadolinium enhancement (LGE), starting at least 5 minutes after contrast administration, and in the same views as the functional study (TR/TE/matrix/segments = 6.2 ms/3.1 ms/ 240 × 210/25). The TI was adjusted to the value required to null the signal from the healthy myocardium. Whenever LGE was seen, the same sequence with swapped phase-encoding direction and different TI was repeated to rule out artifacts.

All the images were analysed with dedicated software (Medis, Leiden, The Netherlands). Analysis was carried out by two observers with at least 10 year experience in CMR. The following variables were quantified: left and right (LV, RV) ventricular end-diastolic (EDV) and end-systolic (ESV) volumes, LV mass, end-diastolic wall thickness, end-diastolic and end-systolic diameters, left atrial (LA) maximum anteroposterior diameter and area. Regional wall motion abnormalities and myocardial edema were investigated. Myocardial perfusion was visually assessed. Localization, pattern of transmural extent and quantification of LGE were obtained. Additionally, aortic and pulmonary trunk dimensions were measured and aortic plaques detected.

A control group of 80 healthy subjects (40 males, 40 females) of the same age deciles as the study subjects (20-60 years of age), belonging to a cohort whose normal values of reference have been previously published elsewhere [14,15], were used as a modelled reference for comparison. Since the healthy controls had been scanned at 1.5 T, we first carried out a comparison test on 8 subjects in which we acquired SSFP cines at both 1.5 T and 3 T, with the same sequence parameters that were eventually used, and in whom ventricular parameters were measured. We obtained a typical interstudy variability of < 4% for all these variables and none of the differences observed between both field strengths were statistically significant. We also demonstrated typical intraclass correlation coefficients for absolute agreement between both analysis softwares > 0.90.

### Statistical analysis

The data obtained were transferred into a database program SPSS 17.0 (IBM, USA). All the study variables were tested for normality with the Kolmogorov-Smirnov test. Continuous variables were expressed as mean ± standard deviation, categorical variables were presented as percentages. Student-t test was used with the continuous Gaussian variables or, alternatively, the Wilcoxon test was employed. Qualitative variables were analyzed with the Chi-square test. Analysis of variance (ANOVA) was used whenever more than two levels of a variable were observed. Logistic regression using stepwise method was employed to analyze the odds ratio (OR) of the severity variables with significant results in the univariate analysis.

## Results

**Figure 1:**
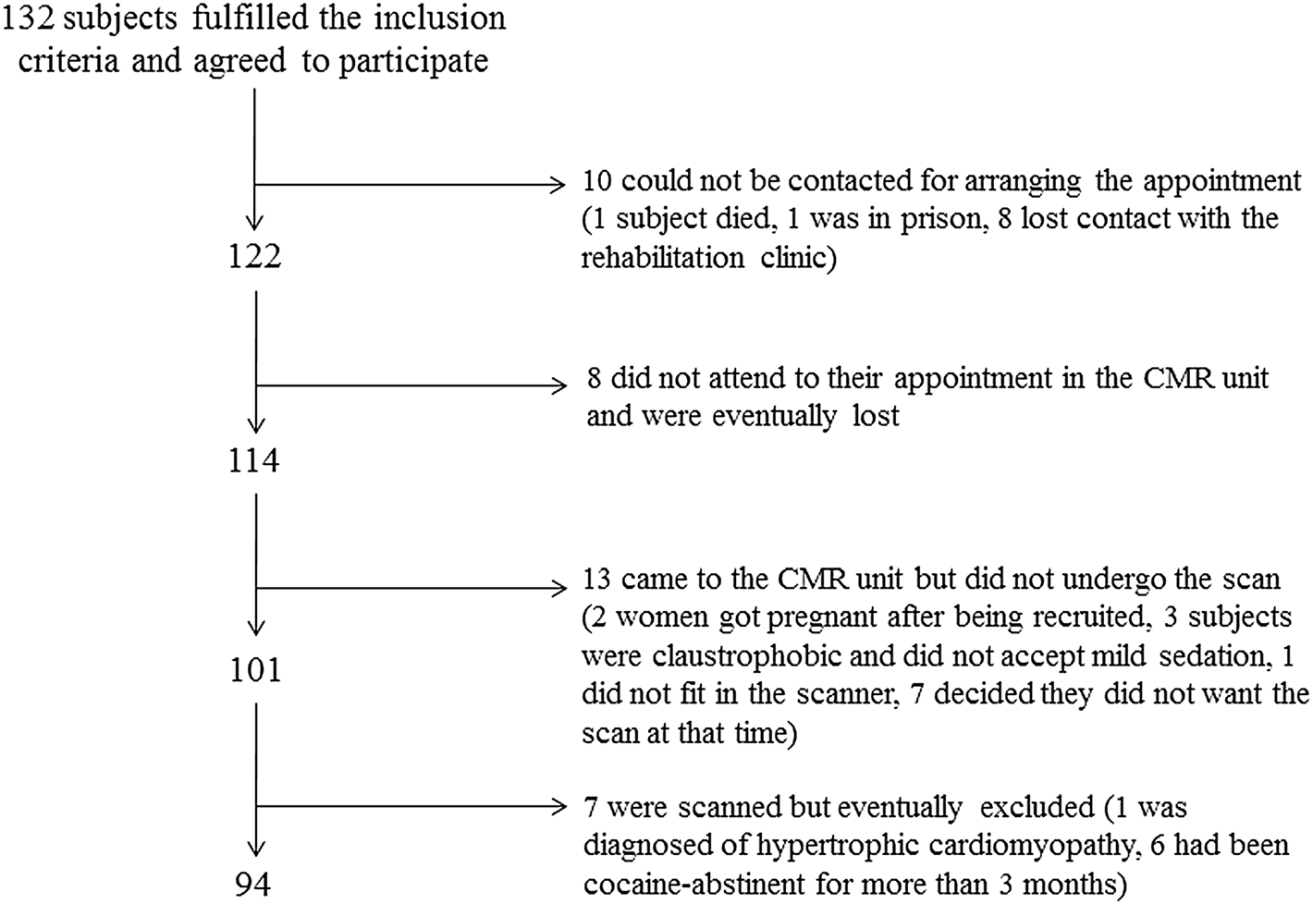
Patient recruitment and final participation in the study.

Initially, 132 CA were recruited. For some reasons (Figure 1) only 94 CA completed the whole protocol and were included in the study (13 females, age range 22-53 yrs, 13.9 ± 9 yrs of addiction). The time interval between the last episode of cocaine uptake and CMR was 53 ±40 days. Data on cocaine and alcohol abuse are presented in Table 2.

**Table 2:** Data on cocaine and alcohol use

Cocaine	Age at the time of first use, yrs	22.7 ± 6.7
	Time between last use and CMR, days	53 ± 40
	Frequency of use in the past (number of uses)	3.2 ± 1.8
	Frequency of use in the past 3 months (uses per month)	3.8 ± 1.6
	Maximum frequency of use in lifetime (uses per month)	7.5 ± 1.5
	Amount of consumption in the past month (g)	3.8 ± 1.6
	Years of regular cocaine use (yrs)	13.9 ± 9
	Route of administration	
	Nasal insufflation	81%
	Smoked	16%
	Intravenous	3%
Alcohol	Subjects with alcohol abuse or dependence, n (%)	49 (51%)
	Age at the time of first use, yrs	16 ± 4
	Time between last use and CMR (days)	120 ± 237
	Frequency of use in the past month (number of uses)	2.7 ± 2.3
	Frequency of use in the past 3 months (uses per month)	2.8 ± 2.3
	Amount of use in the past month (gr)	120 ± 170
	Years of alcohol use	20 ± 7 yrs

Table 3 shows the CA’s biological characteristics and cardiovascular risk factors. No significant differences were found in height, weight and blood pressure between CA and controls. There were 14 CA with no cardiovascular risk factors, and 66, 11 and 3 with 1, 2 and 3 risk factors, respectively. The most prevalent risk factor was current smoking. Only 2 hypertensive CA were receiving therapy with ACE inhibitors. None of the dyslipidemic CA complied with therapy. Laboratory tests are summarised in Table 4. Ultra-sensitive C-reactive protein (usCRP) was significantly elevated in 19% of CA, gammaglutamil transpeptidase (GGT) in 8.5% and fibrinogen in 7.5%. In all cases, urine cocaine metabolites at the time of the scan were negative. Although CA were asymptomatic, 2 of them mentioned palpitations during cocaine use. On examination there were no significant findings except for elevated blood pressure in 4 subjects.

**Table 3:** Biological data and cardiovascular risk factors

Males/females	81/13
Age (yrs)	36.6 ± 7
Heart rate (bpm)	71 ± 12
SBP (mmHg)	127 ± 10
DBP (mmHg)	82 ± 11
Weight (Kg)	77 ± 16
Height (cm)	173 ± 7
Body mass index (Kg/m2)	25.8 ± 4.5
Subjects with overweight, n (%)	27 (28.7%)
Subjects with obesity, n (%)	8 (8.5%)
Smoking habitus	
Currently smoking, n (%)	71 (75%)
Ex–smoker, n (%)	5 (5.3%)
Years of smoking habitus	13.5 ± 9.1
Cigarettes per day	13 ± 7
Dyslipidaemia, n (%)	10 (10.6%)
Years of known dyslipidaemia	1.9 ± 2.0
Hypertension, n (%)	4 (4.2%)
Years of known hypertension	3.6 ± 6.4
Family history of CAD, n (%)	6 (6.3%)

*SBP* systolic blood pressure, *DBP* diastolic blood pressure.

**Table 4:** Laboratory findings

usCRP (mg/L)*	1.8 (0.2 , 32)
subjects increased with usCRP (n, %)	18, 19
Glucose (mg/dL)	79 ± 18
Total cholesterol (mg/dL)	250 ± 42
LDL cholesterol (mg/dL)	127 ± 33
Triglycerides (mg/dL)	244 ± 140
Fibrinogen (mg/dL)	475 ± 45
Gammaglutamiltranspeptidase (IU/L)	79 ± 32
Fosfatase alkaline (IU/L)	159 ± 29

*usCRP is presented as median (minimum, maximum).

ECG abnormality was found in 44 CA (46.8%), which minor repolarization abnormalities such as flat T waves or < 0.5 mm ST depression in 26 patients (27%), significant repolarization abnormalities in the inferior leads in 4 CA and in frontal leads in another 3. ECG criteria of LV hypertrophy (LVH) [28] were present in 7 CA. Finally, 7 CA showed sinus bradycardia, 1 CA had Mobitz 1 second degree AV block and another CA had a short PR interval.

### CMR findings

Intraobserver and interobserver variability were assessed for LV and RV parameters in a subset of 15 CA. Intraobserver variability was below 4% for left ventricular parameters and below 5% for right ventricular parameters. Typical interobserver variabilities were below 5% and 5.5% respectively.

The results of the CMR scans in the CA group were compared to those of the age-matched control group (Table 5). The LVESV was significantly enlarged (30 ± 8 vs 26 ± 5 mL/m^2^, P < 0.01) and the LV mass index was increased (80 ± 13 vs 69 ± 9 g/m^2^, P < 0.05) in the CA group. The relative wall mass was also significantly increased (1.07 ± 0.22 vs 0.87 ± 0.12, P < 0.01). No relation was found between blood pressure levels and LV mass. The LV ejection fraction (EF) was decreased (59 ± 6% vs 67 ± 5%, P < 0.01).

**Table 5:** Per-group analysis of CMR derived parameters

	**Cocaine abusers**	**Controls**	**P**
LVEDVi (mL/m2)	72 ± 15	76 ± 9	NS
LVESVi (mL/m2)	30 ± 8	26 ± 5	< 0.01
LVSVi (mL/m2)	42 ± 9	51 ± 6	< 0.01
LVEF (%)	59 ± 5	68 ± 4	< 0.01
LVMi (g/m2)	76 ± 15	69 ± 4	0.015
RWM (g/mL)	1.07 ± 0.22	0.87 ± 0.12	< 0.01
RVEDVi (mL/m2)	74 ± 16	79 ± 11	NS
RVESVi (mL/m2)	36 ± 9	28 ± 4	< 0.01
RVSV (mL/m2)	42 ± 9	51 ± 7	< 0.001
RVEF (%)	56 ± 5	65 ± 5	< 0.001
LA AP diameter (cm)	31.2 ± 5.1	30.8 ± 4.8	NS
LA area (cm2)	23.5 ± 4.0	24.7 ± 4.0	NS

*LV* left ventricle; *RV* right ventricle; *EDVi* end-diastolic volume index, *ESVi* left ventricular end-systolic volume index, *SVi* stroke volume index, *EF* left ventricular ejection fraction, *LVMi* left ventricular mass index, *RWM* relative wall mass, *LA*: left atrium, *AP* anteroposterior.

With regard to the RV, interestingly and similarly to the LV, the RVESV was significantly increased in the CA group (36 ± 9 vs 28 ± 4 mL/m^2^, P < 0.01). Accordingly, there was a significant decrease in RVEF (56 ± 5% vs 65 ± 5%, P < 0.01).

**Figure 2:**
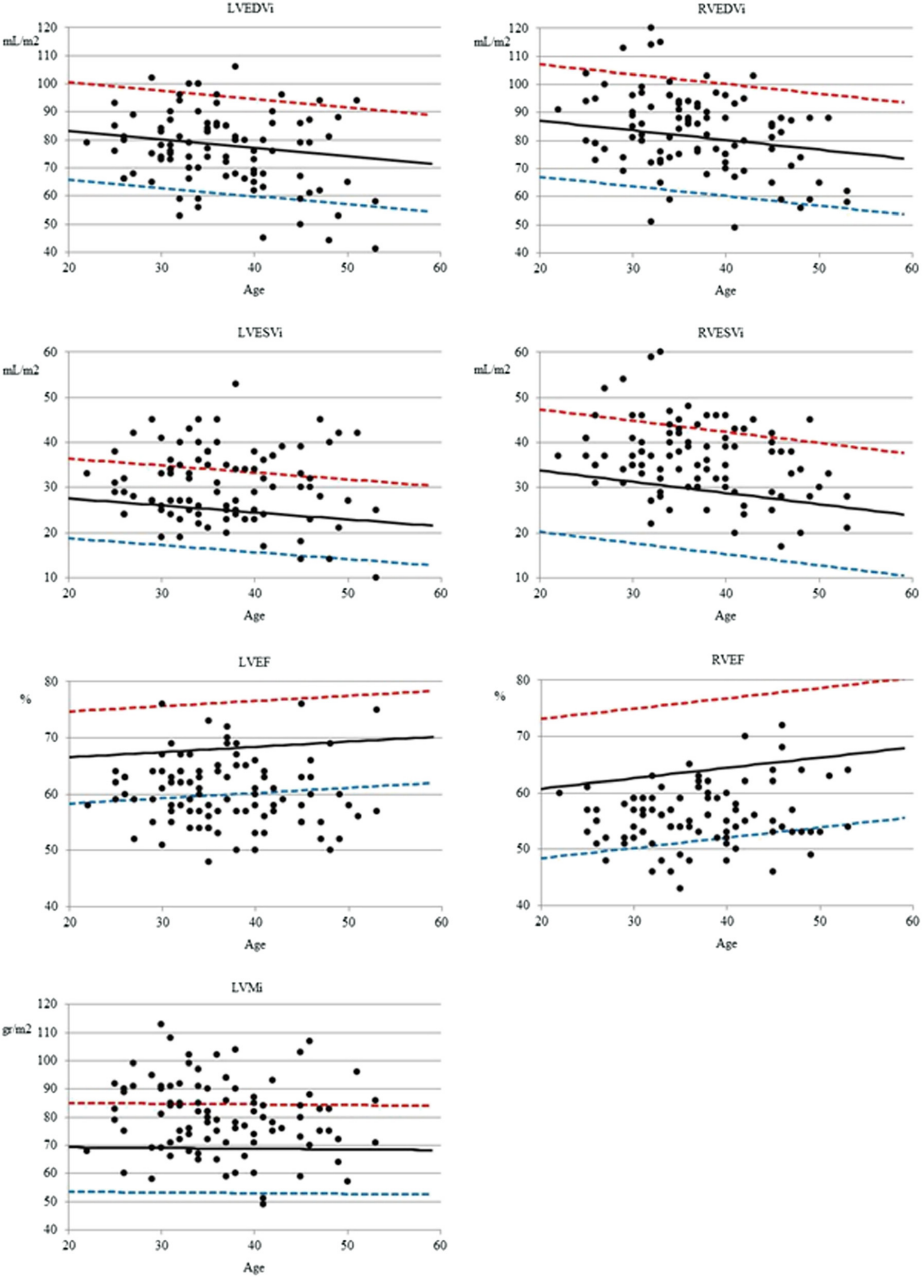
**Per-patient analysis of LV and RV parameters in cocaine abusers (black dots) compared to reference values (mean: black line, red: upper limit of normal, blue: lower limit of normal) showing that a substantial number of asymptomatic cocaine abusers show mild biventricular dilatation and systolic dysfunction, as well as LV hypertrophy.** LV left ventricle; RV right ventricle; EDVi end-diastolic volume index; ESVi, end-diastolic volume index; EF ventricular ejection fraction; LVMi, left ventricular mass index.

**Figure 3:**
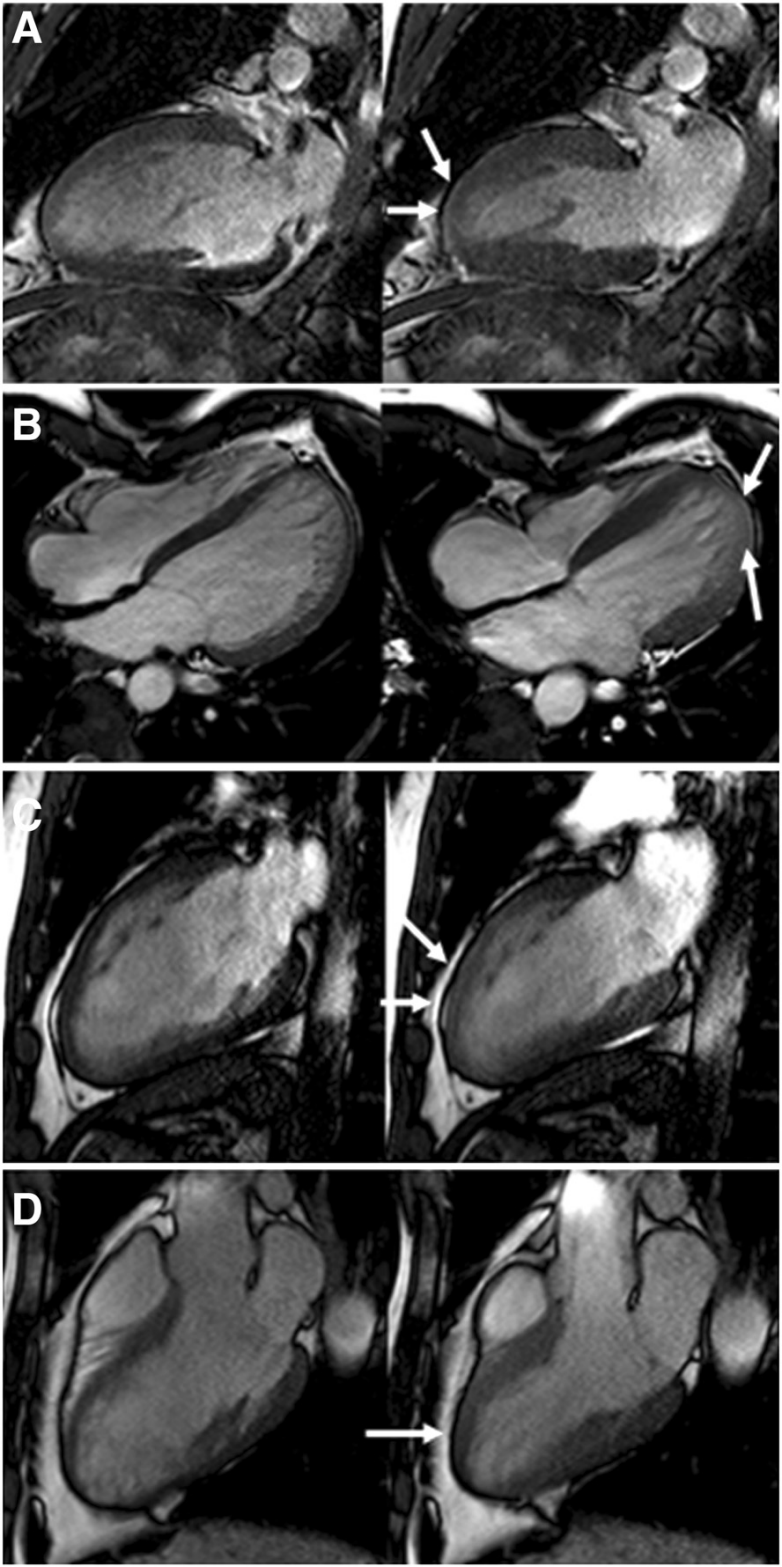
**Regional wall motion abnormalities (arrows) were found in 4 subjects. A)** and **C)** mild anteroapical hypokinesia; **B)** Apicolateral hypokinesia; **D)** distal septum hypokinesia.

In a per-subject analysis, compared to normal controls, 9 CA (10%) had increased LVEDV and 29 CA (31%) had an increased LVESV. Furthermore, 32 CA (34%) showed decreased LVEF, the lowest being 48% (Figure 2, Additional file 1: Video S1). Mild regional wall motion abnormalities were seen in 5 CA, which were located in all cases in the apical segments (Figure 3). With regard to LV morphology, 12 CA (13%) showed concentric remodelling and 28 (30%) showed LVH which was in 15 cases concentric (Additional file 2: Video S2) and in another 13 eccentric (Additional file 3: Video S3). LA dilatation was observed in 7 CA (7.4%), 4 of which had LVH and another 2 LV dilatation. With regard to the RV, 4 CA (4%) had RVEDV above normal values, and as many as 12 (13%) had an RVESV above the normal range. Finally, 16 CA (17%) had decreased RVEF, with the lowest value being 43% (Figure 2, Additional file 4: Video S4). Overall, 13 CA (14%) had biventricular systolic dysfunction (Additional file 5: Video S5).

No CA showed increased myocardial signal intensity indicating oedema on STIR sequences.

In the first 48 subjects dipyridamole stress myocardial perfusion imaging was performed, we observed perfusion defects in 4 patients,

Dipyridamole stress myocardial perfusion images were visually analysed in the first 48 CA. Only 4 CA showed perfusion abnormalities, which in all cases were small, subendocardial, short-lasting and not suggestive of significant epicardial coronary artery disease [29]. These perfusion defects were associated with concentric LVH and concentric LV remodelling in 2 CA, respectively, but not with ventricular dilatation or systolic dysfunction. In the remaining 46 CA, no myocardial perfusion defects were observed at rest.

**Figure 4:**
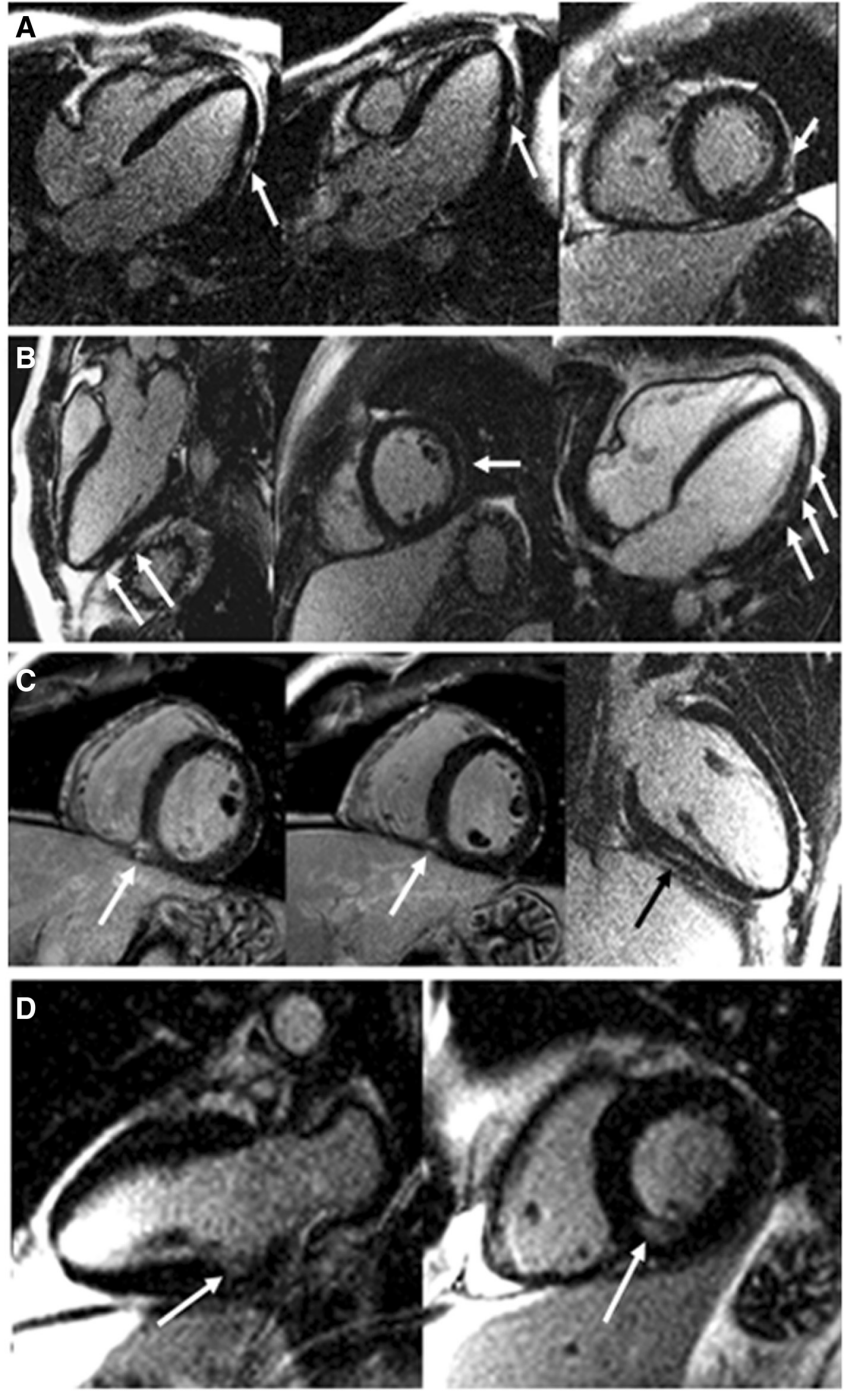
**Late gadolinium enhancement (LGE) in 4 cocaine abusers (arrows). A)** focal subepicardial LGE at the distal mid-lateral wall. **B)** several intramyocardial foci of LGE in the inferolateral and lateral wall. **C)** focal LGE in the mid inferior ventricular junction. **D)** limited subendocardial LGE in the basal inferior wall.

On LGE imaging, 29 CA (30%) showed focal myocardial gadolinium enhancement. LGE extension was very limited, affecting 1 myocardial segment in 27 CA, and 2 segments in the remaining 2 CA. In 1 CA, LGE was subendocardial and located in the inferior wall, in another 2 CA a subepicardial pattern was found, it was midmyocardial in 14, and finally, it was located at the inferior RV insertion point in the remaining 10 CA. The mean percentage of focal myocardial fibrosis was 0.26 ± 0.5% of LV mass (Figure 4). The presence or extent of LGE was not associated with the presence of regional wall motion abnormalities, LV hypertrophy or LV or RV dilatation or systolic dysfunction. Furthermore, the pattern of LGE of the inferior RV insertion point did not appear to be related to dilatation of either the RV or the pulmonary trunk.

Aortic and pulmonary trunk dimensions of CA were compared to published reference ranges [30,31]. Mean aortic sinus diameter was 32±3.5 mm (range 21-40 mm), and mild dilatation of the sinus was observed in 9.6% of CA. Likewise, mean diameter of the ascending aorta was 28.7±4.2 mm (range 21-41 mm), and 8.5% had dilatation of the ascending aorta. Mean diameter of the descending thoracic aorta was 21.5±2.9 mm (range 15-29 mm) with 10.6% of CA showing dilatation of this segment. With regard to the pulmonary trunk, mean diameter at the bifurcation level was 22.7±3.1 mm (range 17-39 mm), and it was shown to be dilated in 6.4% of CA. Finally, T2W FSE sequences also detected small, mural aortic plaques in 7 CA.

### CMR findings and cardiovascular risk factors, laboratory findings, ECG

No differences were found in ventricular parameters according to the presence of cardiovascular risk factors, elevated usCRP, GGT or fibrinogen. Patients who smoked had an OR of 1.109 (1.027, 1.198) for developing aortic plaques. There was a poor correlation between CMR and ECG for detection of LVH. Only 3 patients with LVH on CMR had electrocardiographic criteria of LVH, while 4 patients with electrocardiographic LVH had normal LV mass on CMR. The presence of ECG abnormalities was not associated with a higher percentage of regional wall motion abnormalities, LGE, LVH or dilatation, however there was a higher percentage of patients with LV systolic dysfunction among those with abnormal ECG findings (Χ^2^ = 13.42, p = 0.009) (Figure 4).

### CMR findings and severity of cocaine abuse, way of use

We selected the number of years of regular cocaine use as the most adequate variable to characterize the severity of cocaine abuse. This was tested against CMR findings shown as categorical variables. Only those variables with significant or nearly significant differences in univariable analysis were included in the multivariable analysis.

The numbers of years of regular cocaine use was significantly associated with the presence of LV systolic dysfunction (P < 0.01) and with aortic dilatation (P < 0.05), and borderline significantly for RV systolic dysfunction (P =0.06). Logistic regression showed that each year of regular cocaine use increased the risk of mild LV systolic dysfunction by nearly 10% (OR = 1.08, 95% CI = 1.016-1.15, P < 0.01). No significant differences were found in CMR findings between nasal, intravenous or smoked cocaine users. Likewise, no correlation was found between CMR findings and time since last use.

### CMR findings and concomitant alcohol abuse or dependence

Among cocaine abusers, 51% of subjects had concomitant alcohol abuse or dependence. No significant differences in ventricular dimensions and function were seen according to severity of alcohol consumption.

## Discussion

We have used CMR at 3 T to describe the adverse effects caused by chronic cocaine consumption on the cardiovascular system in asymptomatic CA, compared to a group of healthy subjects, which consist mainly in mild LV dilatation, dysfunction and hypertrophy, RV dysfunction and focal myocardial fibrosis. Importantly, the RV was affected to some degree in nearly 25% of CA. Overall, some degree of cardiac involvement was shown in 71% of CA, and LV dysfunction was related to number of years of regular cocaine use, which may have practical importance in these patients’ medical care. It has been previously shown that cocaine is cardiotoxic in both symptomatic [32] and asymptomatic [13,33] CA, but, as far as we are aware, this is the first comprehensive study to assess with CMR cocaine cardiac and vascular toxicity in a reasonably large cohort of consecutive, non-selected, asymptomatic CA, comparing LV and RV results to healthy age and gender matched controls.

### Cocaine and the left ventricle

We observed mild LV dilatation in 31% of the subjects included. This is in concordance with previous literature [34,35]. Whether this represents cocaine-related cardiomyopathy or ischemic cardiomyopathy remains unclear but in our series no relation was found between LV dilatation and ECG signs of necrosis, or LGE suggestive of previous myocardial necrosis. We also observed global LV systolic dysfunction in 34% of our series. This prevalence was higher than in previous echocardiographic reports on asymptomatic patients, in which it ranged from 0% to 14% [7,13,32,36]. Still, we only found mild systolic dysfunction, with the lowest LVEF being 48%, probably because we assessed the chronic effect of cocaine and not the acute phase effect, and also because our cocaine abusers were asymptomatic and likely to have less severe cardiac involvement. With this study we cannot ascertain the mechanism leading to systolic dysfunction, as this was not our aim. Potential mechanisms have been suggested that could explain cocaine-related LV dysfunction including ischemia, repetitive sympathetic stimulation, direct toxicity, myocarditis, increased oxidative stress or activation of genes associated to apoptosis. We dare suggest that there is no single mechanism but several interacting, plus individual susceptibility.

With regard to LV anatomy, we found a pattern of concentric LV remodelling in 13% of subjects and LV hypertrophy in 30%, which was in 15 cases concentric and in another 13 eccentric, not related to blood pressure levels. This finding may have prognostic value given the different prognosis that each pattern of LVH or remodeling has [37]. Autopsy-based studies assessing cocaine-related LVH usually include a small number of patients and use different reference ranges for diagnosing LVH, so their results are hardly comparable. They usually show a high prevalence of LVH, roughly around 50% [2,38-40], which might reflect a more advanced stage of the disease corresponding to autopsy-based studies, and a different method of measurement. In vivo echocardiographic studies have shown similar results^6^ but in asymptomatic abusers this had not been reported before. Unfortunately, so far no single mechanism has been proven to be responsible for LVH in this setting but some have been suggested, including the presence of frequent hypertensive peaks, direct stimulation of myocardial α-adrenergic receptors, and increased expression of cardiomyocyte proteins mediated by protein kinase-C and calcium/calmodulin kinase II-dependent mechanisms.

### Cocaine and the right ventricle

Another interesting finding in our study is the presence of RV involvement in these subjects. We observed RV dilatation in 13% of subjects. Likewise, RV systolic dysfunction was observed in 17% of subjects, which was in all cases global. Again, though the reason for this remains unclear, similar systemic mechanisms to those suggested for LV dysfunction could probably be applicable to the RV.

### Cocaine, myocardial oedema, perfusion and focal LGE

We did not find signs of acute myocarditis such as increased focal myocardial signal intensity in the STIR sequences. In contrast to our findings, both autopsy [12,38] and clinical studies [13] have shown a significant proportion of myocarditis and oedema, associated with one or more positive urinary assay for cocaine metabolites, which probably indicates a more recent exposure to cocaine.

We could only assess stress myocardial perfusion in 48 CA. Still, we did not find any hypoperfusion pattern suggestive of significant epicardial coronary artery disease but only small, subendocardial, short-lasting perfusion defects in 4 CA, not related to ventricular dilatation or dysfunction in any case. Though invasive coronary angiography was not performed, we hypothesize that these perfusion defects could represent small vessel disease, which is a frequent finding in CA [2].

We found focal myocardial LGE in 30% of our patients. One patient showed a subendocardial pattern suggestive of myocardial necrosis, but not associated with a wall motion abnormality. In another two patients LGE was subepicardial, suggestive of past myocarditis. Finally, it was midmyocardial in 14 and in another 10 it was located in the inferior RV junction. The presence of coronary disease is not a sine qua non for the development of cardiac injury and late gadolinium enhancement. A previous publication in a casual cocaine user has demonstrated multiple spots of midmyocardial LGE taken as potential evidence of cocaine-induced vasoconstriction of coronary arterioles [41]. Furthermore, myocardial infarction can occur as a result of coronary thrombus in patients with otherwise normal coronary arteries [42]. Therefore, some of the midmyocardial areas of LGE found might well account for coronary vasospasm or small areas of thrombus occlusion, bearing in mind that this pattern is also observed in a number of cardiomyopathies.

Finally, we report the unexpected finding of LGE of the inferior RV insertion point in 10 CA. This particular pattern of LGE has been previously associated with adverse outcome in pulmonary hypertension, but we did not find RV wall motion abnormalities or pulmonary trunk dilatation in these patients, though this pattern was as frequent as midmyocardial LGE in patients with RV systolic dysfunction.

### Correlation of CMR findings with cardiovascular risk factors and ECG

We observed ECG abnormalities of variable degree in nearly half of the subjects, similar to other authors [13,43-45], mainly consisting of minor changes. The presence of ECG changes regardless the type, was only associated to a higher proportion of LV systolic dysfunction. We also found a poor correlation between CMR and ECG for detection of LVH, concordant with previously acknowledged limitations of ECG in detecting LVH [46].

### CMR findings and severity of cocaine addiction, way of use

Though it makes sense to quantify the amount of cocaine use as a marker of the severity and duration of cocaine abuse, there is no widely accepted quantitative variable. Cocaine abusers find it very difficult to recall accurately their usage. Also, as cocaine addiction may span many years, the abuse level can vary considerably. We chose number of years of regular cocaine use among a number of potential variables because this is simple for use in these patients, and also because it seemed the most accurate reflecting severity of cocaine abuse. We found that this variable was associated with the likelihood of cardiac involvement, reflecting cocaine cardiotoxicity, which may have practical utility for the overall medical care of these patients.

### CMR findings and concomitant alcohol abuse or dependence

In our study, 51% of cocaine users had concomitant alcohol abuse or dependence. This proportion falls within the published ranges of 30-75% [47,48]. We did not find a relation between concomitant alcohol consumption and increased severity of cardiovascular involvement. This is in contrast with previously reported findings. When combined with ethanol, the concentration of cocaine increases, and the active metabolite cocaethylene is formed which blocks the reuptake of dopamine and helps to strengthen and prolong cocaine cardiovascular side effects [49]. It is not clear why alcohol did not have a further effect on cardiovascular findings in our study, but might arise from differences in our population characteristics and amount of alcohol consumption compared with previous studies.

### Study limitations

Our study population may not be representative of subjects with only a recreational use of cocaine, or those who by their living standards have no financial problems maintaining their addiction who do not suffer economic, health or social problems, and thus may not seek medical care. Although we recruited consecutive patients, there is a potential selection bias as they were referred from the addictions treatment unit, which might indicate a more severe addiction or higher suspicion of heart disease. We cannot rule out coronary artery disease as a potential explanation to our findings as we did not perform relevant diagnostic investigations. We could not compare LGE findings in addicts with those of healthy subjects because we did not perform LGE in controls as that was not the scope of that particular study. Also, echocardiography was not performed so potential causes for RV dysfunction such as pulmonary hypertension could not be ruled out. Still, though 6% of addicts showed mild pulmonary trunk dilatation, we did not observe significant tricuspid regurgitation in the cine sequences in any of them. Finally, though the sample size of our study is one of the largest published to date, it remains fairly limited in power to detect small differences from normal. However, cocaine addicts are a difficult group of patients to study and are not reliable attenders for investigations and research.

## Conclusion

CMR detected cardiovascular disease of variable degree in 71% of this cohort of consecutive cocaine abusers. The main findings were a decrease in systolic function of both left and right ventricles, an increase of left ventricular mass and the presence of focal fibrosis. There was a significant association between years of cocaine use and probability of LV systolic dysfunction.

## Competing interests

The authors declare that they have no competing interests.

## Authors’ contributions

AMM: conception and design, data collection, analysis and interpretation of data, drafting of the manuscript, final approval. CR: conception and design, data collection, drafting of the manuscript, final approval. JC-S: design, critical review of the manuscript, final approval. BI: design, data collection, critical review of the manuscript, final approval. MG: design, data collection, drafting of the manuscript, final approval. JS: conception and design, analysis and interpretation of data, critical review, final approval. VB: conception and design, critical review, final approval. DP: conception and design, interpretation of data, critical review of the manuscript, final approval.

## Supplementary Material

Additional file 1 **Video S1.** Cine sequence in the 4-chamber view of a CA showing mild left ventricular systolic dysfunction. LVEF was 49%. Click here for file

Additional file 2 **Video S2.** Cine sequence in the 4-chamber view of a CA with concentric LVH. LVMi was 96 g/m2 and the relative wall mass was 1.18. Click here for file

Additional file 3 **Video S3.** Cine sequence in the 4-chamber view of a CA with eccentric LVH. LVMi was 95 g/m2 and the relative wall mass was 0.91. Click here for file

Additional file 4 **Video S4.** Cine sequence in the 4-chamber view of a CA showing right ventricular systolic dysfunction. RVEF was 44%. Click here for file

Additional file 5 **Video S5.** Cine sequence in the 4-chamber view of a CA showing mild biventricular systolic dysfunction. LVEF was 50% and RVEF was 46%. Click here for file
